# Molecular phylogeny of *Salmo* of the western Balkans, based upon multiple nuclear loci

**DOI:** 10.1186/1297-9686-46-7

**Published:** 2014-02-03

**Authors:** Gašper Pustovrh, Aleš Snoj, Simona Sušnik Bajec

**Affiliations:** 1 Department of Animal Science, Biotechnical Faculty, University of Ljubljana, Groblje 3, SI-1230 Domžale, Slovenia

## Abstract

**Background:**

Classification of species within the genus *Salmo* is still a matter of discussion due to their high level of diversity and to the low power of resolution of mitochondrial (mt)DNA-based phylogeny analyses that have been traditionally used in evolutionary studies of the genus. We apply a new marker system based on nuclear (n)DNA loci to present a novel view of the phylogeny of *Salmo* representatives and we compare it with the mtDNA-based phylogeny.

**Methods:**

Twenty-two nDNA loci were sequenced for 76 individuals of the brown trout complex: *Salmo trutta* (Danubian, Atlantic, Adriatic, Mediterranean and Duero mtDNA lineages), *Salmo marmoratus* (marble trout), *Salmo obtusirostris* (softmouth trout), and *Salmo ohridanus* (Ohrid belvica or belushka). Sequences were phylogenetically analyzed using maximum-likelihood and Bayesian Inference methods. The divergence time of the major clades was estimated using the program BEAST.

**Results:**

The existence of five genetic units i.e. *S. salar*, *S. ohridanus*, *S. obtusirostris*, *S. marmoratus* and the *S. trutta* complex, including its major phylogenetic lineages was confirmed. Contrary to previous observations, *S. obtusirostris* was found to be sister to the *S. trutta* complex and the *S. marmoratus* clade rather than to the *S. ohridanus* clade. Reticulate evolution of *S. obtusirostris* was confirmed and a time for its pre-glacial origin suggested. *S. marmoratus* was found to be a separate species as *S. trutta* and *S. obtusirostris*. Relationships among lineages within the *S. trutta* complex were weakly supported and remain largely unresolved.

**Conclusions:**

Nuclear DNA-based results showed a fairly good match with the phylogeny of *Salmo* inferred from mtDNA analyses. The comparison of nDNA and mtDNA data revealed at least four cases of mitochondrial–nuclear DNA discordance observed that were all confined to the Adriatic basin of the Western Balkans. Together with the well-known extensive morphological and genetic variability of Balkan trouts, this observation highlights an interesting and variegated evolutionary history of *Salmo* in this area.

## Background

Due to the high level of phenotypic diversity recorded in trout species, the classification of the genus *Salmo* is still a matter of discussion. According to a recent taxonomic evaluation [1], about 30 species have been identified, which is in marked contrast to the two-species view (e.g. [2]), which was held for many years and which recognized only *S. trutta* and *S. salar* (e.g. [3,4]). Genetically, representatives of the genus *Salmo*, with the exception of *S. salar*, are usually regarded as members of the *Salmo trutta* complex, which includes five mitochondrial (mt)DNA lineages named after the basins where they were first found: Danubian (DA), Atlantic (AT), Adriatic (AD) and Mediterranean (ME) [5], and the drainage Duero (DU; [6]). A separate lineage, *marmoratus* (MA), corresponds to the marble trout (*S. marmoratus*) sampled in the Po and Soča river drainages. Although a consensus has been reached on the existence of these different phylogeographic lineages, the relationships among the lineages are unclear and result in different interpretations of their evolution (e.g. [7-10]). Moreover, a number of studies have shown that some lineages are also naturally present outside of their described geographical range. For example, the AD and ME lineages are present respectively in the Iberian Peninsula [8] and in the Adriatic basin [11], while the AT lineage has been found in Sicily [12] and is also apparently naturally present in the Danubian drainage in northern Austria [13]. In addition, MA haplotypes have been detected in brown trout [10,11] and AD haplotypes in the southern population of marble trout, from the Neretva and Skadar (*Shkodra* in Albanian) basins [14].

From a taxonomic view, mtDNA lineages have not been validated, with the exception of *marmoratus*, despite the inconsistency reported for marble trout, which is regarded either as a separate species (e.g., [11,15-17]) or a member of the *S. trutta* complex [7,18].

Besides *S. salar* and the *S. trutta* complex, there are two less well known but phenotypically highly distinct members of *Salmo*: *S. ohridanus* (Ohrid belvica; *belushka* in Albanian) and *S. obtusirostris* (softmouth trout), both of which have been ambiguously classified despite their distinct morphology, and their position within Salmonidae has been rearranged on numerous occasions (see Snoj et al. [19] for review). On the basis of molecular data, these two species have been positioned in the genus *Salmo*[20-22]. While *S. ohridanus* has turned out to be undisputedly sister to the *S. trutta* complex, the exact position of *S. obtusirostris* has yet to be resolved; depending on the molecular markers used and model of evolution applied, *S. obtusirostris* is assigned either to the *S. trutta* complex or as sister to *S. ohridanus*[19,23].

Although several other *Salmo* species have been described from the Balkans [1], the systematics of the majority of these species is still uncertain [24]. Therefore, in addition to *S. trutta* and *S. salar*, we will refer to the taxonomic classification of only those taxa for which separation has been previously described at the molecular level i.e. *S. marmoratus*, *S. obtusirostris*, *S. ohridanus*; for the other taxa, we use the designation brown trout *Salmo trutta* complex.

Some phylogeny studies of *Salmo* have also included nuclear (n)DNA single loci but were either subsequently reported to be phylogenetically non-informative due to selection pressure (*transferrin*[25,26]; *ITS*[27]) or screened in only a few *Salmo* taxa (lactate dehydrogenase LDH-C1*[19]; *growth hormone*[28]; *ITS1*[20]) with no intention of resolving their phylogenetic position within the genus. Phylogeny studies using other sequences of nDNA in *Salmo* have not been undertaken and, therefore, no comprehensive nDNA information is available to verify the conclusions based on the analysis of the control region (CR) mtDNA.

The discrepancy that exists between gene trees and a species tree, and also between nDNA and mtDNA trees, is well known [29-31] and is especially problematic in closely related species or those with large population sizes [32], a situation commonly observed in *Salmo*. With the development of novel techniques, it is now becoming easier to collect data on multiple unlinked nuclear gene loci and multiple individuals per species [32-34]. In addition, new analytical methods have emerged to evaluate species trees, based either on concatenated datasets and previously used methods to construct phylogenies (maximum likelihood, parsimony, Bayesian analyses) or on the coalescent theory, which analyzes genetic loci individually and constructs a species tree from the results of independent analyses of individual loci [32].

In order to resolve phylogeographic and taxonomic inconsistencies in *Salmo*, predominantly in trouts of the Western Balkans, and to avoid misinterpretations due to possible discrepancies between gene and species trees, we have applied a new marker system based on a larger number of independent and neutral nDNA loci than that previously used and which are designed to distinguish purebred trouts from their hybrids [35].

## Methods

### Samples and DNA isolation

**Figure 1:**
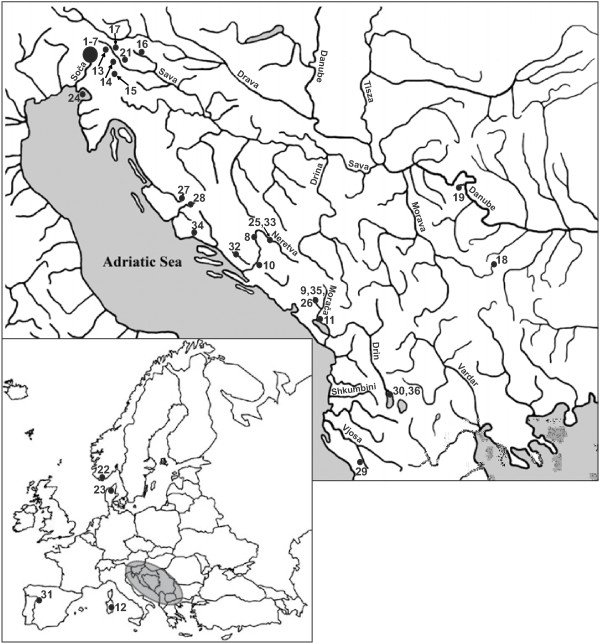
**Map of locations.** Map of Europe and the western Balkans showing the locations of the 36 sampling sites. Sampling site numbers correspond to those (NS) in Table 1.

A total of 76 individuals were analyzed (Table 1, Figure 1), including marble trout *S. marmoratus* (northern and southern populations, N = 20), brown trout *S. trutta*, (N = 42, phylogeographic lineages DA, AT, AD, ME and DU, hereafter referred to as *S. trutta* complex), Ohrid belvica (belushka) *S. ohridanus* (N = 2), softmouth trout *S. obtusirostris* (from the rivers Neretva, Vrljika, Jadro and Zeta, N = 8) and as out-groups Atlantic salmon *S. salar* (N = 2) and Sakhalin Taimen *Parahucho perryi* (N = 2). Sequence data from 36 individuals (18 marble trout, 12 brown trout, four dentex and two Atlantic salmon) were recovered from a previous study ([36]; for Genbank accession numbers see Table 2). Data on the phylogeographic lineage and introgression status of all analyzed samples were obtained from mtDNA and microsatellite markers tests in previous studies [15,23,37,38].

**Table 1:** Sample information

**Species (common name)**	**mtDNA phylogenetic lineage**	**Location (country)**	**NS**	**N**	**OTU**
*Salmo marmoratus* (mt)	MA	Predelca (SLO)	1	2	1
*S. marmoratus* (mt)	MA	Zadlaščica (SLO)	2	2	1
*S. marmoratus* (mt)	MA	Studenc (SLO)	3	2	1
*S. marmoratus* (mt)	MA	Trebuščica (SLO)	4	2	1
*S. marmoratus* (mt)	MA	Idrijca (SLO)	5	2	1
*S. marmoratus* (mt)	MA	Lipovšček (SLO)	6	2	1
*S. marmoratus* (mt)	MA	Huda grapa (SLO)	7	2	1
*S. marmoratus* (mt)	AD	Neretva (SLO)	8	2	2
*S. marmoratus* (mt)	AD	Zeta (MNE)	9	2	2
*S. dentex* (zubatak)	AD	Neretva (BIH)	10	2	2
*S. dentex* (zubatak)	AD	Zeta (MNE)	11	2	3
*S. trutta* (bt)	ME	Sardinia (I)	12	2	4
*S. trutta* (bt)	DA	Ribnica (SLO)	13	2	5
*S. trutta* (bt)	DA	Sovpot (SLO)	14	2	5
*S. trutta* (bt)	DA	Mahnečica (SLO)	15	2	5
*S. trutta* (bt)	DA	Kremžarjev potok (SLO)	16	2	5
*S. trutta* (bt)	DA	Mošenik (SLO)	17	2	5
*S. trutta* (bt)	DA	Studena (SRB)	18	2	5
*S. trutta* (bt)	DA	Vratovina (SRB)	19	2	5
*S. trutta* (bt)	AT	Mount Lassen-hatchery (USA)	20	2	6
*S. trutta* (bt)	AT	Povodje-hatchery (SLO)	21	2	6
*S. trutta* (bt)	AT	Otra (NOR)	22	2	6
*S. trutta* (bt)	AT	Denmark-hatchery (DK)	23	2	6
*S. trutta* (bt)	AT	Adriatic Sea (SLO)	24	2	6
*S. trutta* (bt)	AD	Neretva (BIH)	25	2	3
*S. trutta* (bt)	AD	Zeta (MNE)	26	2	3
*S. trutta* (bt)	AD	Zrmanja (HR)	27	2	3
*S. trutta* (bt)	AD	Krka (HR)	28	2	3
*S. trutta* (bt)	AD	Bistrica (ALB)	29	2	3
*S. trutta* (bt)	AD	Lake Ohrid (FYROM)	30	2	3
*S. trutta* (bt)	DU	Duero (ESP)	31	2	7
*S. obtusirostris* (st)		Vrlika (HR)	32	2	8
*S. obtusirostris* (st)		Neretva (BIH)	33	2	9
*S. obtusirostris* (st)		Jadro (HR)	34	2	8
*S. obtusirostris* (st)		Zeta (MNE)	35	2	8
*S. ohridanus* (belvica/belushka)		Lake Ohrid (FYROM)	36	2	10
*S. salar* (Atlantic salmon)		Denmark-hatchery (DK)		2	11
*Parahucho perryi* (Taimen)		Koppi (RUS)		2	12

Species name, mtDNA phylogenetic lineage, location of sampling site, number of sampling sites (NS), number of individuals sampled per location (N) and operational taxonomic units used in *BEAST analyses (OTU); SLO = Slovenia, MNE = Montenegro, BIH = Bosnia and Herzegovina, I = Italy, SRB = Serbia, USA = United States of America, NOR = Norway, DK = Denmark, HR = Croatia, ALB = Albania, FYROM = Former Yugoslav Republic of Macedonia, RUS = Russia, ESP = Spain; common species names: mt = marble trout, bt = brown trout, st = softmouth trout.

**Table 2:** Locus information

**Locus**	**Genbank accession numbers**	**jModeltest BIC**	**MrModeltest AIC**	**Ktreedist**
GP1	HM066793, HM066804-HM066818, HM635370-HM635372, KG699502-KG699507	TrN	GTR	0.04589
GP4	HM066821, HM066825, HM635375-HM635376, KG699508-KG699509	F81	F81	0.04593
GP5	HM066826-HM066827, HM066830-HM066832, HM635378-HM635379	JC	HKY	0.04555
GP14	HM635380-HM635384	JC	F81	0.03149
GP16	HM066835, HM066841-HM066842, HM635386, KG699510-KG699511	F81	F81	0.04473
GP31	HM066845-HM066846, HM635387, KG699512-KG699513	K80 + G	HKY	0.02257
GP34	HM066852-HM066861, HM635389-HM635391, KG699514-KG699515	HKY	HKY + I	0.04559
GP37	HM066863, HM066869-HM066873, KG699516-KG699518	HKY	HKY	0.04048
GP38	HM066880, HM066882, HM635392-HM635393, KG699519-KG699520	JC	JC	0.04485
GP42	HM066884-HM066885, HM066888-HM066890, HM635395, KG699521-KG699523	F81	F81	0.04562
GP73	HM066893, HM066898-HM066901, HM635396KG699525	F81 + G	F81	0.04062
GP81	HM635402, HM635405-HM635409, HM635411-HM635412, KG699526	F81 + G	F81	0.04583
GP85	HM066916-HM066925, HM635415-HM635418, KG699527-KG699533	F81	GTR + G	0.04133
GP94	HM066928-HM066934, HM635420, KG699534-KG699536	F81	HKY + I	0.04585
HMG1	HM066734, HM066737, HM635469, KG699537-KG699541	F81	HKY + I	0.02706
SS2	HM066740-HM066745, HM635458-HM635460, KG699542	F81	HKY + I	0.04591
TFGB-beta	HM066759, HM066761-HM066765, HM635472-HM635473, KG699543	TIMef	F81 + I	0.02028
tnfa	HM066769, HM066781-HM066784, HM635474-HM635476, KG699544-KG699547	TrN + G	HKY + I	0.02342
RH	HM635423, HM635425-HM635430, HM635432-HM635433, KG699548-KG699549	F81	F81	0.04299
SILVA	HM635434-HM635435, HM635437, HM635440-HM635444, HM635446, KG699550-KG699553	JC	HKY	0.04580
SL	HM635451, HM635455-HM635457, KG699554-KG699560	JC	GTR + I	0.03979
TF	HM635463-HM635467, HM635469-HM635470, KG699561-KG699562	JC	HKY	0.02376

Locus name, GenBank accession numbers of amplified sequences, nucleotide substitution model for each partition (locus) and differences between gene trees and species tree (Ktreedist).

DNA was isolated from fin clips conserved in 96% ethanol, using the high-salt extraction technique of Miller et al. [39].

### Sequencing

Twenty-two nuclear regions were analyzed. Polymerase chain reaction (PCR) primers and conditions are described in Pustovrh et al. [36] and PCR were performed in 25 μL reaction mixtures according to [35]. Amplified DNA fragments were run on a 1.5% agarose gel and purified using the QIAEX II Gel Extraction Kit (QIAGEN). Approximately 100 ng of purified PCR product was used in cycle sequencing reactions following BigDye Terminator v3.1 Cycle Sequencing protocols (Applied Biosystems). The amplified, fluorescently labeled and terminated DNA was salt-precipitated and analyzed with an ABI 3130 XL Genetic Analyzer. All newly determined sequences were submitted to Genbank (see Table 2).

### Alignment, data partitioning and phylogenetic analysis

Sequences of all 22 loci for each individual sample were combined in the same order as reported in Table 2 and aligned using the default parameters in CLUSTAL-W [40]. Genotypes of all loci in each of the analyzed samples are reported [See Additional file 1: Table S1].

All loci were tested for positive selection (HA: dN > dS) by the Nei–Gojobori method [41] using MEGA version 4 [42].

A phylogenetic tree based on all the individuals analyzed was constructed using maximum-likelihood (ML) and Bayesian Inference (BI) methods. ML was performed as implemented in GARLI Version 0.96b8 [43]. To avoid over partitioning and yet still effectively deal with heterogeneity, each locus was used as a criterion to define a partition (locus). Prior model selection for each partition was determined using the Bayesian information criterion (BIC) calculated in jMODELTEST v 3.06 [44] in conjunction with PAUP (models for each partition are listed in Table 2). For analysis, 2000 bootstrap replicates were carried out to identify the best partitioning scheme. Analysis was performed with the settings recommended by Zwickl [43] and the runs were set for an unlimited number of generations and automatic termination following 20,000 generations without a meaningful (ln L increase of 0.01) change in score.

For BI, MrBayes 3.1.2 [45] was used. Prior model selection for each partition was determined using the Akaike Information Criterion (AIC) calculated in MrModeltest 2.3 [46] in conjunction with PAUP (Table 2). Random starting trees were used and four Markov chains were run for one million generations: nucmodel = 4 by 4; nruns = 2; tree-sampling frequencies of 1 in 100 (10,000 trees saved). Convergence was assessed by examining the cumulative posterior probabilities of clades using the “Are We There Yet?” online program (AWTY; [47]).

To address inter-specific phylogenetic relationships within *Salmo*, the method for species tree reconstruction, implemented in *BEAST v1.7.2. [34], was also used. The program determines the likelihood of gene trees in a given species tree to find the most likely containing species tree for multiple genes sampled from individuals across a set of closely related species. It uses the Markov chain Monte Carlo approach to average over the tree space, so that each tree is weighted proportionally to its posterior probability. All settings were entered in BEAUti v1.7.0, a graphical software package that allows creation of BEAST XML input files. Each genetic region (locus) was partitioned using BIC calculated in jMODELTEST v 3.06 (Table 2). Time-measured phylogeny using the relaxed clock model [48] and tree model were set as unlinked for all partitions. All molecular clock estimates for the gene regions were examined using the uncorrelated lognormal model. The operational taxonomic units (OTU) that were compared corresponded with the clades defined on the basis of individual trees constructed in GARLI as described above (see Table 1). Using CIPRES Science Gateway V. 3.3 [49], a public resource for inference of large phylogenetic trees, the same XML file was run for seven independent simultaneous runs of 300 millions generations sampled once every 30,000 trees. After analyzing each run separately, the program Tracer v1.5 (distributed with BEAST) was used and the first 30 millions generations were discarded as burn-in samples. A summary tree was created for each run using TreeAnnotator v1.5.3 (distributed with BEAST). The separate log and tree files were combined using LogCombiner v1.5.3 (distributed with BEAST). The combined log file was analyzed in Tracer 1.5 to ensure that effective sample sizes were large enough. Combined trees were analyzed with TreeAnnotator 1.5.3 and a summary tree produced and viewed in FigTree version 1.3.1 (http://tree.bio.ed.ac.uk/software/figtree/).

Each gene tree generated with BEAST was also analyzed and compared to the reference species tree. This was assessed by K tree score implemented in the program Ktreedist [50] that measures overall differences in the relative branch length and topology of two phylogenetic trees (two trees with very different relative branch lengths will get a high K score while two trees that follow a similar between-lineage rate variation will get a low K score, regardless of the overall rates in both trees; by comparing two trees you can choose the one that better follows the overall shape of a given reference tree [50].

### Divergence date estimation

The time of divergence between major clades within *Salmo* since they last shared a common ancestor was estimated using the Relaxed Bayesian molecular clock model in BEAST v.1.7.2. The first prior was chosen to set the time to the most recent common ancestor (TMRCA) of *S. ohridanus* and *S. trutta* complex, which, on the basis of Cyt*b* sequences was estimated to be 4 MYA (million years ago) [22] (prior was set to 4 MY (million years) with standard deviation (SD) = ±1 MY.) Another prior was chosen to set the time when the DA and AD–ME clades last shared a common ancestor. It is generally considered that the Mediterranean and Danubian basins separated about 700,000 years ago, and thus the prior was set to 0.7 MY with SD = ±0.2 MY [7,51].

All the other settings were the same as for the *BEAST analysis described above and median values and 95% highest posterior density intervals of the corresponding TMRCA were obtained. The results were analyzed with Tracer v 1.5.

## Results

### Alignment and loci properties

Final alignment was made for 76 samples and 22 nuclear loci (Tables 1 and 2). Some loci could not be amplified in the out-group species (four in *S. salar* and 10 in *P. perryi*). When excluding the out-group species, the final alignment consisted of ca. 8000 bp with 234 variable sites, 196 of which were parsimony-informative. All DNA sequences have been deposited in GenBank (accessions numbers are in Table 2).

None of the loci analyzed proved to be under selection pressure according to the Nei–Gojobori method.

All the gene trees produced by *BEAST analysis were concordant and their topology and shape were similar to those of the reference species tree, as reflected by a low K score below 0.05 in the 22 comparisons (Table 2). Thus, none of the genes was excluded from the *BEAST input file and all gene trees were used to calculate the species tree.

### Phylogenetic analysis

**Figure 2:**
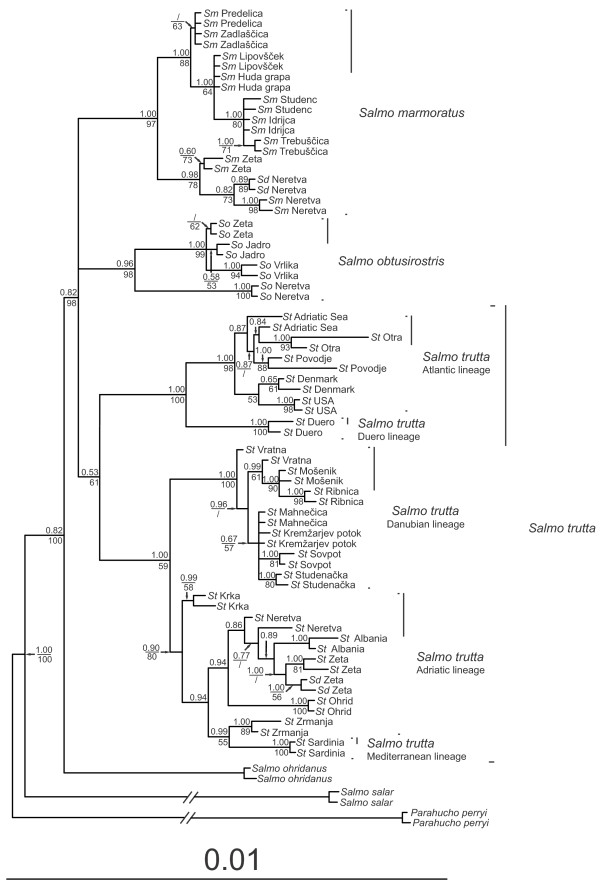
**Phylogenetic tree of genus *Salmo *calculated with GARLI and MrBayes.** Posterior probabilities are written above, and ML bootstrap values below, each branch. Branches and values less than 0.50/50% are not plotted. Sm, *S. marmoratus*; Sd, *S. dentex*; So, *S. obtusirostris*; St, *S. trutta*.

The topologies of the phylogenetic trees based on individuals using either the ML or BI method were very similar (Figure 2) with two main branches: one branch corresponded to *S. ohridanus* and the other to a cluster of three groups of trouts that included northern and southern populations of marble trout and its dentex ecotype, softmouth trout, and a complex of brown trout lineages. Since these three groups split from the same internal node (polytomy), their evolutionary relationship could not be resolved.

The complex of brown trout lineages included two sister groups: one originating from the Atlantic basin and the other from the Danubian and Mediterranean basins. The split between these two groups was only moderately supported (MrBayes posterior probability (MrB PP) = 0.53; ML bootstrap value (ML BS) = 0.61). The Atlantic basin group was further split into two clades that corresponded to the AT and DU mtDNA phylogeographic lineages, while the Danubian and Mediterranean group formed two clades that corresponded to the DA and AD-ME mtDNA phylogeographic lineages. In the latter clade, AD and ME lineages could not be clearly distinguished i.e. individuals from the Zrmanja River (Adriatic basin) bearing AD haplotypes showed a sister relationship to individuals from Sardinia (Mediterranean lineage) bearing ME haplotypes.

**Figure 3:**
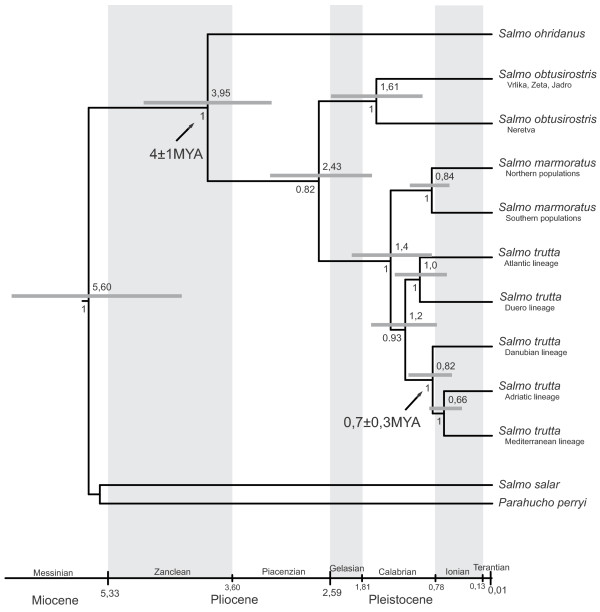
Phylogenetic tree of species or lineages of genus *Salmo *calculated using the Bayesian *BEAST method. Posterior probabilities for each split are indicated below, and time of separation from most common ancestor (in million years, MYA) and confidence intervals above, each branch. The priors used for calculation of time of split from most common ancestor are marked with an arrow.

The relationships among OTU shown on the phylogenetic tree based on species and lineages using the Bayesian *BEAST method (Figure 3) were similar to those on the tree in Figure 2 but with a few specificities i.e. (1) instead of polytomy for marble trout, softmouth trout and brown trout, a split between softmouth trout and a group consisting of two sister clades (marble trout and brown trout) was observed, although with a low posterior probability in BEAST (BE PP = 0.82) and a high statistical support in ML (98%) and (2) within the brown trout complex, a split between individuals from the Atlantic and the Danubian and Mediterranean basins was supported with a high probability (BE PP = 0.93) like the split between individuals from the Adriatic basin and from Sardinia (BE PP = 1).

### Divergence date estimates

Based on the output of *BEAST and with the two priors of 4 ±1 MYA for a split between *S. trutta* and *S. ohridanus*, and 0.7 ±0.2 MYA, when the DA and AD–ME clades last shared a common ancestor, as well as a relaxed clock, the time of the most recent common ancestor was estimated for each sister pair in Figure 3. Accordingly, the divergence date between Atlantic salmon and the trout complex was estimated in the late Miocene, between softmouth trout and the other trouts (brown and marble) in the late Pliocene, and between marble trout and the brown trout complex in the mid-Pleistocene about 1.5 MYA. For details on the divergence among brown trout and other intra-species, see Figure 3.

## Discussion

### Phylogenetic relationship

Previously, phylogenetic analyses of the genus *Salmo* were based primarily on variations within CR mtDNA and in general, the resolution of the relationships among OTU was difficult. Furthermore, many examples confirm that a phylogeny based on mtDNA does not necessarily reflect the accepted phylogeny of the species. This is expected when dealing with species that have evolved recently as is the case of *Salmo*. For example, mtDNA analyses of *S. obtusirostris salonitana* (Jadro softmouth trout) and *S. obtusirostris zetenzis* (Zeta softmouth trout) have revealed haplotypes that are typical of the brown trout AD lineage rather than those that are normally present in softmouth trout. It is only after microsatellite analysis that mtDNA capture (reticulate evolution) was shown to be involved in both populations ([52]; unpublished data). Whereas mtDNA describes only the maternal history of populations, nuclear markers always reflect maternal and paternal gene flow and allele histories, and are therefore more appropriate to reconstruct species tree. By using a new system based on 22 nuclear loci, we have improved the phylogeny of *Salmo* and checked its match with the CR mtDNA-based phylogeny.

Overall, our results show a fairly good match with the phylogeny of *Salmo* inferred from CR mtDNA, since several identical genetically homogeneous groups are found with both approaches. We confirm the existence of at least five distinct genetic units: Atlantic salmon, Ohrid belvica, softmouth trout, marble trout and the brown trout complex, all of which can be distinguished from each other. However, our results do not support the sister relationship between Ohrid belvica and softmouth trout as previously hypothesized based on external morphology [53] and mtDNA genetics [19]. Instead, our results point to a greater affinity of softmouth trout with the brown trout complex and marble trout. However, since these three species originate from one node, their relationships remain unresolved (Figures 2 and 3). We found that softmouth trout from the rivers Jadro and Zeta, despite having Adriatic haplotypes, cluster together with other softmouth trout in the same clade, which confirms the previously reported suggestion of reticulate evolution for this species [52].

The fact that softmouth trout is a spring spawner suggests that it evolved prior to the Pleistocene as a consequence of an evolutionary conservatism related to water temperature during spawning (contrary to glacial species such as brown and marble trout, which require colder water and thus spawn in winter; for details, see Karaman [54]). A pre-glacial origin for softmouth trout is also confirmed by the divergence time estimated from *BEAST analysis (Figure 3). However, it is important to emphasize the somewhat unreliable statistical support for this divergence time, although molecular markers specific to softmouth trout have never been found outside of the species’ present range and are exclusive to the narrow strip of the Western Balkans. Thus, both ecological and genetic observations imply that the origin of softmouth trout lies in this area, and that its distribution has remained limited to the Western Balkans since the time of its origin (ca. late Pliocene) until today.

According to the mtDNA-based phylogeny, *S. marmoratus* is a separate lineage within the *S. trutta* complex (*marmoratus*, MA [7]), and closely related to the AD lineage. Both lineages are considered to have originated in the Italo-Adriatic and Balkans at a time when the ME lineage evolved in the western Mediterranean (reviewed in Bernatchez [7] and Cortey et al. [8]). However, we reposition *S. marmoratus* on the basis of the nDNA results reported here that classify it as a distinct cluster of the same or similar rank as that of *S. trutta* and *S. obtusirostris*. This observation supports the classification of marble trout as a distinct species; indeed, this is the most notable specificity that differentiates the nDNA-based tree topology (“species tree”) presented here from the previously constructed mtDNA-based trees.

Our study reveals the genetic divergence between the northern and southern populations of marble trout, which could not be previously seen because of the polyphyletic origin of mtDNA haplotypes of these populations. It also provides new insight into the genetic structure of *S. marmoratus* with a considerably higher level of genetic polymorphism than previously reported on the basis of mtDNA studies [7]. For example, the genetic structure of seven populations of marble trout in Slovenia that are geographically separated and represent a resource for repopulation of the species [16] has been well studied both with mtDNA ([23,55]; unpublished data) and microsatellite markers [15]. Whereas the mtDNA analysis was poorly informative due to the fixation of a single haplotype (MA1a) that suggested the absence of population substructuring, microsatellite analysis revealed a very strong inter-population genetic differentiation (pairwise fixation index (F_ST_) from 0.3-0.9) probably because of a combination of a high level of microsatellite mutation and random drift (all the populations are very small). Analysis of the nDNA of the seven Slovenian populations grouped them into four genetically homogeneous units, their geographic distribution coinciding logically with the spatial proximity of the corresponding populations; according to our results, the populations diverged in the late Pleistocene. Thus, there is evidence that the marker system reported here is sufficiently polymorphic and informative to establish times of divergence for trout lineages within *Salmo*.

The least well supported branching on the tree concerns the *S. trutta* complex (Figures 2 and 3) for which the progress of how brown trout lineages developed is unclear. The lowest statistical significance was found for the relationship between OTU within the Mediterranean–Adriatic clade, but this poor resolution might be due to the small number of Mediterranean individuals sampled. However, the development of these lineages runs across a relatively narrow timeframe, as suggested both by the *BEAST-TMRCA analysis and by Bernatchez [7], which could be the most likely reason for such a low phylogenetic resolution in the rest of the *S. trutta* complex in this study too. Our results suggest that the Atlantic group was the first brown trout lineage to split off. Ancestral divergence in the AT lineage has been demonstrated at the mtDNA level [7], and also by the existence of a nucleotide polymorphism in the *ITS* nuclear gene [27] but this was not confirmed in later studies (e.g., mtDNA analyses [8-10], and *transferrin* nuclear gene analyses [8-10]), which considered the DA lineage as the oldest. We found that the DA lineage was sister to the ME–AD lineages, which, according to TMRCA, split around 0.66 MYA as previously estimated from variation in mtDNA [7]. The early mid-Pleistocene (0.7 MYA) saw the most drastic paleohydrological changes in the last three MY that led to the separation of previously connected river basins [56]. Taking into account both the nDNA and mtDNA data, the separation of the Danubian basin from the Adriatic basin seems to be one of the most marked hydrological changes that occurred at that time.

### Mitochondrial–nuclear discordance

**Figure 4:**
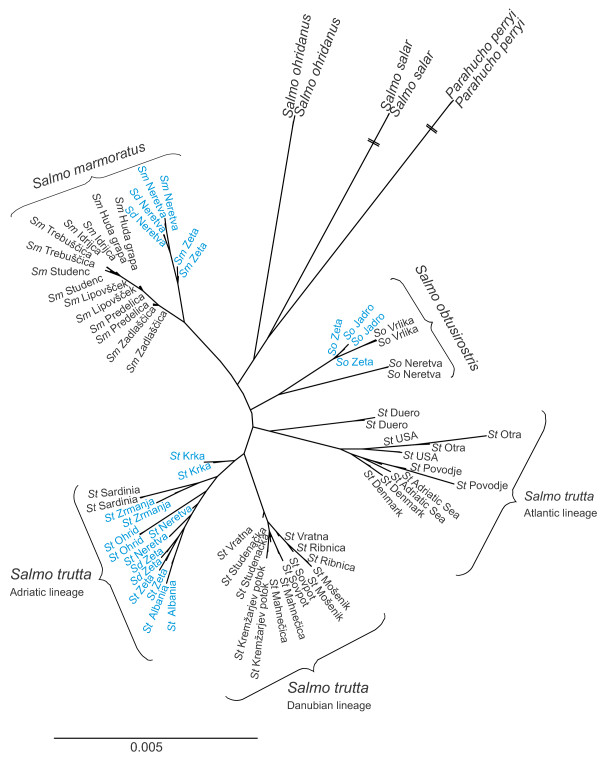
**Mitochondrial–nuclear discordance in *Salmo *evidenced through a star-shaped phylogenetic tree obtained by maximum likelihood.** Samples characterized by AD mt-phylogenetic lineage are marked in blue. Sm, *Salmo marmoratus*; Sd, *Salmo dentex*; St, *Salmo trutta.*

Comparison of nDNA and mtDNA data detected at least four cases of mitochondrial–nuclear discordance for *Salmo* (in *S. marmoratus* and *S. obtusirostris*; Figure 4), three of which had been previously reported based on microsatellite, or single gene analyses, or both, and mtDNA (*S. obtusirostris salonitana*[52]; *S. obtusirostris zetensis*[37]; *S. marmoratus*[36]). We also observed a new case of mitochondrial–nuclear discordance within softmouth trout with the population from the Vrljika River bearing a Neretva softmouth trout-specific mtDNA haplotype [21] although it clustered with the Jadro and Zeta populations in the same clade on the basis of nDNA data (Figures 2 and 4). Interestingly, all reported cases of mitochondrial–nuclear discordance in *Salmo* are limited to the Adriatic river systems in the Western Balkans. Such discordances have been reported for other salmonids (*Oncorhynchus*[57] and *Salvelinus*[58-60]) but to our knowledge, have not been detected in *Salmo* outside the above-mentioned area. The extensive morphological and genetic variability in *Salmo*, together with the large proportion of mitochondrial–nuclear discordance detected in the Adriatic basin of the Western Balkans, point to an interesting and variegated evolutionary history of *Salmo* taxa in the area.

### Note on taxonomy aspects

*Salmo* trouts have undergone an evolutionary radiation in relatively recent times (from mid to late Pleistocene). For this reason, it has been difficult to genetically distinguish the most recently evolved populations, particularly in the Balkans, where trout have evolved into a variety of different forms. Despite this difficulty, the data reported here suggest a clear separation of the species and populations analyzed into several main evolutionary lineages, and further geographical subdivisions of these lineages. Thus, the phylogenetic trees presented here lend support to nomenclature, not only with regard to the species status of *S. ohridanus*, *S. obtusirostris* and *S. marmoratus*, but also as a means for naming the main lineages by names that already exist. Thus, *S. trutta* would apply to the AT lineage and *S. labrax* to the DA lineage, as already proposed [1]. The situation is not so clear for the AD–ME lineage, which comprises the most complicated conglomerate of taxa e.g. *S. macrostigma*, *S. cettii*, and *S. farioides*. However, several meaningful phylogenetic subdivisions have been found in trout from the rivers Krka and Zrmanja that correspond completely with the distribution of the questionable species *S. visovacensis* Taler, 1950 and *S. zrmanjenzis* Karaman, 1938. Similarly, the clear separation of Ohrid brown trout from the other trouts that inhabit nearby areas (River Neretva, the Skadar–Ohrid river system and River Bistrica in southern Albania) implies an independent taxonomic status for the Ohrid brown trout and provides support to maintaining its taxonomic epithet *S. letnica* as previously proposed [37].

Geographically clearly defined sub-lineages are evident also in marble trout. Since these share a similar phylogenetic hierarchical level to that of the main evolutionary lineages of *S. trutta*, they should be regarded as two distinct species. However, no morphological analysis has been undertaken to compare these lineages and they do not show any visible phenotypic differences. The notable exception is *S. dentex*, which appears to be a life-history form of Neretva marble trout [14]. Therefore, further research is necessary to determine whether marbled trout can be split into two geographically separated sister species. For such a classification, a detailed morphological analysis is required and to date, we propose that each lineage is considered as an evolutionary significant unit.

## Competing interests

The authors declare that they have no competing interests.

## Authors’ contributions

GP carried out the molecular genetic studies and analyzed the data. SSB and AS conceived the study, participated in its design and wrote the manuscript. All authors read and approved the final manuscript.

## Supplementary Material

Additional file 1: Table S1 Complete genotypes of the samples analyzed. Location, mtDNA lineage and genotype for each SNP locus analysed is given for all the samples. Click here for file

## References

[B1] Kottelat M Freyhof J Handbook of European Freshwater Fishes 2007 Switzerland and Berlin, Germany: Cornol

[B2] Delling B Species diversity and phylogeny of *Salmo* with emphasis on southern trouts (Teleostei, Salmonidae) PhD Thesis 2003 Stockholm: Stockholm University, Department of Zoology

[B3] MacCrimmon HR Marshall TL World distribution of brown trout *Salmo trutta* J Fish Res Board Can 1968 25 2527 2548 10.1139/f68-225

[B4] Elliott JM Wild brown trout *Salmo trutta–*an important national and international resource Freshwater Biol 1989 21 1 5 10.1111/j.1365-2427.1989.tb01343.x

[B5] Bernatchez L Guyomard R Bonhomme F DNA sequence variation of the mitochondrial control region among geographically and morphologically remote European brown trout *Salmo trutta* populations Mol Ecol 1992 1 161 173 10.1111/j.1365-294X.1992.tb00172.x 1344992

[B6] Machordom A Suarez J Almodovar A Bautista JM Mitochondrial haplotype variation and phylogeography of Iberian brown trout populations Mol Ecol 2000 9 1325 1338 10.1046/j.1365-294x.2000.01015.x 10972772

[B7] Bernatchez L The evolutionary history of brown trout (*Salmo trutta* L.) inferred from phylogeographic, nested clade, and mismatch analyses of mitochondrial DNA variation Evolution 2001 55 351 379 11308093 10.1111/j.0014-3820.2001.tb01300.x

[B8] Cortey M Pla C Garcia-Marin JL Historical biogeography of Mediterranean trout Mol Phylogenet Evol 2004 33 831 844 10.1016/j.ympev.2004.08.012 15522807

[B9] Marić S Sušnik S Simonović P Snoj A Phylogeographic study of brown trout from Serbia, based on mitochondrial DNA control region analysis Genet Sel Evol 2006 38 411 430 10.1186/1297-9686-38-4-411 16790230 PMC2689293

[B10] Snoj A Marić S Berrebi P Crivelli AJ Shumka S Sušnik S Genetic architecture of trout from Albania as revealed by mtDNA control region variation Genet Sel Evol 2009 41 22 10.1186/1297-9686-41-22 19284692 PMC3225828

[B11] Splendiani A Giovannotti M Cerioni PN Caniglia ML Caputo V Phylogeographic inferences on the native brown trout mtDNA variation in central Italy Ital J Zool 2006 73 179 189 10.1080/11250000600679751

[B12] Schöffmann J Sušnik S Snoj A Phylogenetic origin of *Salmo trutta* L 1758 from Sicily, based on mitochondrial and nuclear DNA analyses Hydrobiologia 2007 575 51 55 10.1007/s10750-006-0281-2

[B13] Weiss S Schlötterer C Waidbacher H Jungwirth M Haplotype (mtDNA) diversity of brown trout *Salmo trutta* in tributaries of the Austrian Danube: massive introgression of Atlantic basin fish–by man or nature? Mol Ecol 2001 10 1241 1246 10.1046/j.1365-294X.2001.01261.x 11380880

[B14] Snoj A Glamuzina B Razpet A Zablocki J Bogut I Lerceteau-Köhler E Pojskić N Sušnik S Resolving taxonomic uncertainties using molecular systematics: *Salmo dentex* and the Balkan trout community Hydrobiologia 2010 651 199 212 10.1007/s10750-010-0297-5

[B15] Fumagalli L Snoj A Jesenšek D Balloux F Jug T Duron O Brossier F Crivelli AJ Berrebi P Extreme genetic differentiation among the remnant populations of marble trout (*Salmo marmoratus*) in Slovenia Mol Ecol 2002 11 2711 2716 10.1046/j.1365-294X.2002.01648.x 12453253

[B16] Crivelli A Poizat G Berrebi P Jesenšek D Rubin JF Conservation biology applied to fish: the example of a project for rehabilitating the marble trout (*Salmo marmoratus*) in Slovenia Cybium 2000 24 211 230

[B17] Berrebi P Povž M Jesenšek D Cattaneo-Berrebi G Crivelli AJ The genetic diversity of native, stocked and hybrid populations of marble trout in the Soča river, Slovenia Heredity 2000 85 277 287 10.1046/j.1365-2540.2000.00753.x 11012732

[B18] Meraner A Baric S Pelster B Dalla Via J Trout (*Salmo trutta*) mitochondrial DNA polymorphism in the centre of the marble trout distribution area Hydrobiologia 2007 579 337 349 10.1007/s10750-006-0479-3

[B19] Snoj A Melkić E Sušnik S Muhamedagić S Dovč P DNA phylogeny supports revised classification of *Salmothymus obtusirostris* Biol J Linnean Soc 2002 77 399 411 10.1046/j.1095-8312.2002.00130.x

[B20] Phillips RB Matsuoka MP Konon I Reed KM Phylogenetic analysis of mitochondrial and nuclear sequences supports inclusion of *Acantholingua ohridana* in the genus *Salmo* Copeia 2000 2 546 550

[B21] Snoj A Bogut I Sušnik S Evidence of a genetically distinct population of Vrljika softmouth trout *Salmo obtusirostris* Heckel evolved by vicariance J Fish Biol 2008 72 1945 1959 10.1111/j.1095-8649.2008.01816.x

[B22] Sušnik S Knizhin I Snoj A Weiss S Genetic and morphological characterization of a Lake Ohrid endemic, *Salmo* (*Acantholingua*) *ohridanus* with a comparison to sympatric *Salmo trutta* J Fish Biol 2006 68 2 23 10.1111/j.0022-1112.2006.00902.x

[B23] Razpet A Sušnik S Jug T Snoj A Genetic variation among trout in the River Neretva basin, Bosnia and Herzegovina J Fish Biol 2007 70 94 110 10.1111/j.1095-8649.2007.01392.x

[B24] Delling B Diversity of western and southern Balkan trouts, with the description of a new species from the Louros River, Greece (Teleostei: Salmonidae) Ichthyolog Explor Freshwaters 2010 21 331 344

[B25] Rozman T Dovč P Marić S Kokalj-Vokač N Erjavec-Škerget A Rab P Snoj A Evidence for two transferrin loci in the *Salmo trutta* genome Anim Genet 2008 39 577 585 10.1111/j.1365-2052.2008.01768.x 18786157

[B26] Antunes A Templeton AR Guyomard R Alexandrino P The role of nuclear genes in intraspecitic evolutionary inference: genealogy of the transferrin gene in the brown trout Mol Biol Evol 2002 19 1272 1287 10.1093/oxfordjournals.molbev.a004188 12140239

[B27] Presa P Pardo BG Martinez P Bernatchez L Phylogeographic congruence between mtDNA and rDNA ITS markers in brown trout Mol Biol Evol 2002 19 2161 2175 10.1093/oxfordjournals.molbev.a004041 12446808

[B28] Oakley TH Phillips RB Phylogeny of salmonine fishes based on growth hormone introns: Atlantic (*Salmo*) and Pacific (*Oncorhynchus*) salmon are not sister taxa Mol Phylogenet Evol 1999 11 381 393 10.1006/mpev.1998.0599 10196079

[B29] Maddison WP Gene trees in species trees Syst Biol 1997 46 523 536 10.1093/sysbio/46.3.523

[B30] Hudson RR Coyne JA Mathematical consequences of the genealogical species concept Evolution 2002 56 1557 1565 12353748 10.1111/j.0014-3820.2002.tb01467.x

[B31] Brito P Edwards SV Multilocus phylogeography and phylogenetics using sequence-based markers Genetica 2009 135 439 455 10.1007/s10709-008-9293-3 18651229

[B32] Heled J Drummond AJ Bayesian inference of species trees from multilocus data Mol Biol Evol 2010 27 570 580 10.1093/molbev/msp274 19906793 PMC2822290

[B33] Carstens BC Knowles LL Estimating species phylogeny from gene-tree probabilities despite incomplete lineage sorting: an example from *Melanoplus grasshoppers* Syst Biol 2007 56 400 411 10.1080/10635150701405560 17520504

[B34] Drummond AJ Suchard MA Xie D Rambaut A Bayesian phylogenetics with BEAUti and the BEAST 1.7 Mol Biol Evol 2012 29 1969 1973 10.1093/molbev/mss075 22367748 PMC3408070

[B35] Pustovrh G Sušnik Bajec S Snoj S A set of SNPs for *Salmo trutta* and its application in supplementary breeding programs Aquaculture 2012 370 102 108

[B36] Pustovrh G Sušnik Bajec S Snoj S Evolutionary relationship between marble trout of the northern and the southern Adriatic basin Mol Phylogenet Evol 2011 59 761 766 10.1016/j.ympev.2011.03.024 21440648

[B37] Sušnik S Snoj A Wilson IF Mrdak D Weiss S Historical demography of brown trout (*Salmo trutta*) in the Adriatic drainage including the putative *S. letnica* endemic to Lake Ohrid Mol Phylogenet Evol 2007 44 63 76 10.1016/j.ympev.2006.08.021 17046289

[B38] Jug T Berrebi P Snoj A Distribution of non-native trout in Slovenia and their introgression with native trout populations as observed through microsatellite DNA analysis Biol Conserv 2005 123 381 388 10.1016/j.biocon.2004.11.022

[B39] Miller SA Dykes DD Polesky HF A simple salting out procedure for extracting DNA from human nucleated cells Nucleic Acids Res 1988 16 1215 10.1093/nar/16.3.1215 3344216 PMC334765

[B40] Thompson JD Higgins DG Gibson TJ CLUSTAL-W: improving the sensitivity of progressive multiple sequence alignment through sequence weighting, position-specific gap penalties and weight matrix choice Nucleic Acids Res 1994 22 4673 4680 10.1093/nar/22.22.4673 7984417 PMC308517

[B41] Nei M Gojobori T Simple methods for estimating the numbers of synonymous and nonsynonymous nucleotide substitutions Mol Biol Evol 1986 3 418 426 3444411 10.1093/oxfordjournals.molbev.a040410

[B42] Tamura K Dudley J Nei M Kumar S MEGA4: molecular evolutionary genetics analysis (MEGA) software version 4.0 Mol Biol Evol 2007 24 1596 1599 10.1093/molbev/msm092 17488738

[B43] Zwickl DJ Genetic algorithm approaches for the phylogenetic analysis of large biological sequence datasets under the maximum likelihood criterion PhD thesis 2006 Austin: The University of Texas

[B44] Posada D jModelTest: phylogenetic model averaging Mol Biol Evol 2008 25 1253 1256 10.1093/molbev/msn083 18397919

[B45] Ronquist F Huelsenbeck JP MrBayes 3: Bayesian phylogenetic inference under mixed models Bioinformatics 2003 19 1572 1574 10.1093/bioinformatics/btg180 12912839

[B46] Nylander JAA Ronquist F Huelsenbeck JP Nieves-Aldrey JL Bayesian phylogenetic analysis of combined data Syst Biol 2004 53 47 67 10.1080/10635150490264699 14965900

[B47] Nylander JAA Wilgenbusch JC Warren DL Swofford DL AWTY (are we there yet?): a system for graphical exploration of MCMC convergence in Bayesian phylogenetics Bioinformatics 2008 24 581 583 10.1093/bioinformatics/btm388 17766271

[B48] Drummond AJ Ho SYW Phillips MJ Rambaut A Relaxed phylogenetics and dating with confidence PLoS Biol 2006 4 e88 10.1371/journal.pbio.0040088 16683862 PMC1395354

[B49] CIPRES http://www.phylo.org/index.php/portal/

[B50] Soria-Carrasco V Talavera G Igea J Castresana J The K tree score: quantification of differences in the relative branch length and topology of phylogenetic trees Bioinformatics 2007 23 2954 2956 10.1093/bioinformatics/btm466 17890735

[B51] Webb T Bartlein PJ Global changes during the last three million years: climatic controls and biotic responses Annu Rev Ecol Syst 1992 23 141 173

[B52] Sušnik S Weiss S Odak T Delling B Treer T Snoj A Reticulate evolution: ancient introgression of the Adriatic brown trout mtDNA in softmouth trout *Salmo obtusirostris* (Teleostei : Salmonidae) Biol J Linnean Soc 2007 90 139 152 10.1111/j.1095-8312.2007.00717.x

[B53] Berg LS Vorläufige Bemerkungen über die europäischasiatischen Samoniden, insbesondere die Gattung Thymallus Annuaire du Musée Zoologique de l’Académie Impériale des Sciences de St Petersbourg 1908 500 514

[B54] Karaman S Les salmonidés des Balkans Bull Soc Sci Skoplje 1927 2 258 268

[B55] Snoj A Jug T Melkič E Sušnik S Pohar J Dovč P Jesenšek D Budihna N Mitochondrial and microsatellite DNA analysis of marble trout in Slovenia J Freshwater Biol (Quaderni ETP) 2000 29 5 11

[B56] Georgiev S On the origin of Balkan peninsula Salmonids Ribarstvo, Zagreb 2003 61 147 174

[B57] Miyahara H Yamada H Sato T Harada Y Yamamoto S Kawamura K Mitochondrial-nuclear discordance in the Amago salmon, *Oncorhynchus masou ishikawae*, in the River Miya, Japan Conserv Genet 2012 13 1343 1353 10.1007/s10592-012-0378-2

[B58] Glémet H Blier P Bernatchez L Geographical extent of Arctic char (*Salvelinus alpinus*) mtDNA introgression in brook char populations (*S. fontinalis*) from eastern Quebec, Canada Mol Ecol 1998 7 1655 1662 10.1046/j.1365-294x.1998.00494.x

[B59] Wilson CC Bernatchez L The ghost of hybrids past: fixation of arctic charr (*Salvelinus alpinus*) mitochondrial DNA in an introgressed population of lake trout (*S. namaycush*) Mol Ecol 1998 7 127 132 10.1046/j.1365-294x.1998.00302.x

[B60] Redenbach Z Taylor EB Evidence for historical introgression along a contact zone between two species of char (Pisces: Salmonidae) in northwestern North America Evolution 2002 56 1021 1035 12093016 10.1111/j.0014-3820.2002.tb01413.x

