# Correction: Molecular phylogeny of *Salmo* of the western Balkans, based upon multiple nuclear loci

**DOI:** 10.1186/1297-9686-46-21

**Published:** 2014-04-23

**Authors:** Gašper Pustovrh, Aleš Snoj, Simona Sušnik Bajec

**Affiliations:** 1 Department of Animal Science, Biotechnical Faculty, University of Ljubljana, Groblje 3, SI-1230 Domžale, Slovenia

## Correction

After publication of this work [1], we noted that the same mistake occurs in Table one, Figure one, Figure two, Figure four and on page 6 (Result section). In these places of the article, we wrote that the two brown trout samples of the Mediterranean (ME) lineage came from Sardinia, which is not the case. These two samples of the ME lineage were taken from the small river Caranca, a tributary of the Tet River in France and this lineage carried a single mtDNA haplotype MEcs3. Samples were provided by Patrick Berrebi.
